# A Case of Inflammatory Pseudotumor of the Liver Mimicking Hepatocellular Carcinoma on EOB-MRI and PET

**DOI:** 10.1155/2013/594254

**Published:** 2013-05-27

**Authors:** Hiroyoshi Iguchi, Hitoshi Yamazaki, Hidekazu Tsunoda, Yoshihito Takahashi, Hiroaki Yokomori

**Affiliations:** ^1^Department of Radiology, Kitasato University Medical Center, Saitama, Japan; ^2^Department of Pathology, Kitasato University Medical Center, Saitama, Japan; ^3^Department of Surgery, Kitasato University Medical Center, Saitama, Japan; ^4^Department of Internal Medicine, Kitasato University Medical Center, Saitama, Japan

## Abstract

A 71-year-old man was referred to us for investigation of a liver mass and adenomyomatosis of gallbladder. Findings on ethoxybenzyl diethylenetriamine-enhanced MRI (EOB-MRI) led to a presumptive diagnosis of a 1.5 cm hepatocellular carcinoma (HCC) in the right posterior lobe of the liver. Transcatheter arterial chemoembolization and radiofrequency ablation of the tumor were attempted. After 2 months, CT scan and EOB-MRI showed that the tumor had enlarged to 3 cm. Positron emission tomography (PET) confirmed abnormal metabolic activity with a high standardized uptake value of 7.3 in the lesion. These findings could indicate malignancy such as well-differentiated HCC or cholangiocarcinoma or a benign lesion such as hepatic abscess. Histopathological examination of a liver biopsy revealed a granuloma with many inflammatory cells, leading to a diagnosis of inflammatory pseudotumor of the liver. We report a rare case of hepatic inflammatory pseudotumor with enhancement on EOB-MRI and increased uptake on PET, mimicking HCC.

## 1. Introduction

Inflammatory pseudotumor (IPT) is a rare benign tumor that may develop in various organs [1]. However, IPT of the liver is very rare and is often accompanied by fever, malaise, and abdominal pain. Due to the lack of characteristic clinical and radiological features for diagnosis, IPT may be mistaken as malignant tumor such as cholangiocarcinoma, or liver abscess [1–3]. In particular, IPT may be a vascular-rich tumor demonstrating radiological findings similar to those of hepatocellular carcinoma (HCC) [4]. In a patient with risk factors for HCC, such as chronic viral hepatitis and liver cirrhosis, the presence of IPT may be misdiagnosed as HCC, and a correct diagnosis is established only by biopsy or surgery [5, 6]. This lesion is usually diagnosed clinically or by conventional imaging methods [3, 4]. Clinical diagnosis is difficult in patients with nonspecific symptoms and no serological evidence of inflammation. Laparoscopy and/or open hepatectomy are often performed unnecessarily under a misdiagnosis of HCC or cholangiocarcinoma [5]. Hence, diagnosis of IPT by percutaneous needle biopsy is important to avoid unnecessary exploratory laparotomy or hepatectomy. Unfortunately, needle biopsy does not always rule out malignancy or provide a definitive diagnosis of IPT of the liver [7]. There are a few reports of hepatic IPT diagnosed by fluorine-18 fluorodeoxyglucose positron emission tomography (FDG-PET) [8–10]. 

We herein report a case of IPT of the liver in a patient with cirrhosis, with imaging features mimicking HCC.

## 2. Case Report

A 71-year-old Japanese man was referred to our out-patient clinic in 2012 for investigation of a 1.5-cm liver mass and adenomyomatosis of gallbladder, which were detected incidentally by ultrasonography (US). 

The patient had no history of medication. Physical examination was normal. No stigmata for chronic liver disease was identified. Superficial lymph nodes were not palpable. The lungs were normal on auscultation. A chest radiograph and an electrocardiogram were normal. The results of biochemical analysis of the blood were total bilirubin, 0.8 mg/dL (normal range: 0.2–1.2); direct bilirubin, 0.1 mg/dL (normal range: 0.0–0.2); aspartate aminotransferase (AST), 45 IU/L (normal range: 10–40); alanine aminotransferase (ALT), 50 IU/L (normal range: 5–45); alkaline phosphatase, 419 IU/L (normal range: 100–325); *γ*-guanosine triphosphate, 242 IU/L (normal range: <30), CEA, 4.1 ng/mL (normal range: <5.0); CA19-9, 23.2 U/mL (normal range: <37.0); alpha fetoprotein (AFP), 11.3 ng/mL (normal range: <10.0); protein induced by vitamin K absence or antagonists-II (PIVKA-II), 11 ng/mL (normal range: <10.0). His daily alcohol intake was over 80 g for 40 years. Hepatitis B surface antigen, hepatitis B e-antigen, and hepatitis C virus antibody were negative. 

**Figure 1:**
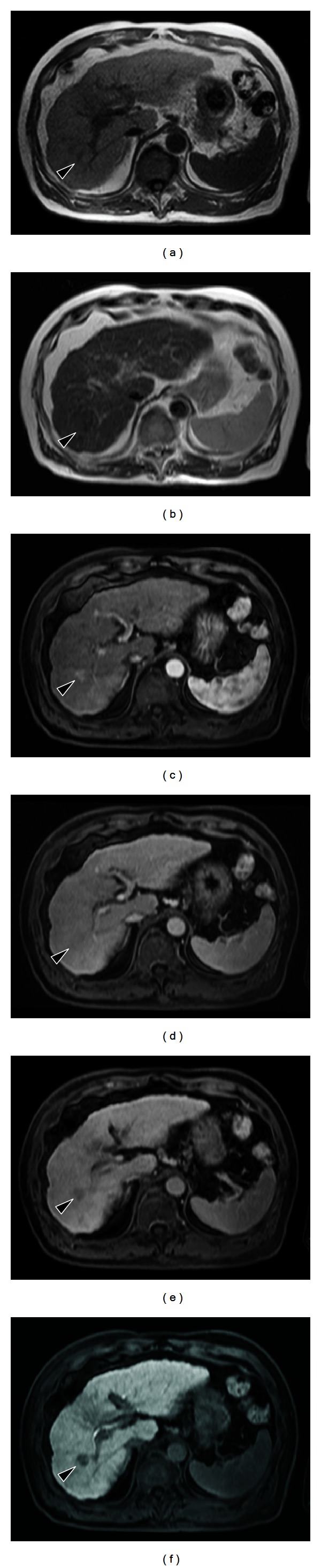
MR images of hepatic inflammatory pseudotumor. (a) The lesion appears isodense (arrow) on T1-weighted image. (b) A partially round, hyperintense lesion (arrow) is observed on T2-weighted image. ((c)–(f)) Gadolinium ethoxybenzyl diethylenetriamine pentaacetic acid-enhanced images. In the arterial phase following contrast injection (c), the center of the tumor is enhanced intensely and homogeneously. In the delayed phase (d) and hepatobiliary phase (f), the tumor is completely washed out. (e) portal phase.

**Figure 2:**
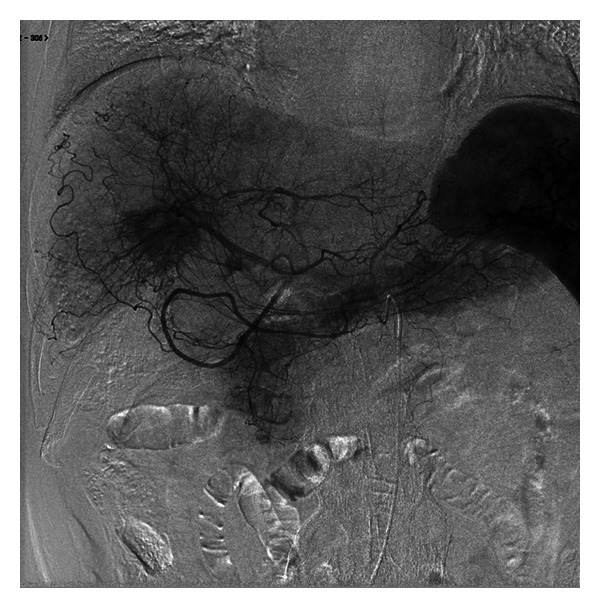
Radiological study of intervention for hepatic inflammatory pseudotumor. Positive staining of nodules (arrows) is visible in segmental 6. The arrowhead denotes tumor staining.

Ultrasonography reviewed a lesion in the liver, which was isoechoic to surrounding parenchyma. Computed tomography (CT) showed a 1.5-cm mass in the right posterior lobe of the liver, with contrast enhancement in early phase and washout with rim enhancement in delayed phase, together with definite findings of cirrhosis such as loss of hepatic volume or hypertrophy of the left liver and caudate lobe. The imaging findings thus indicate cirrhosis with IPT. The etiology might be alcoholic liver injury judging from his alcohol intake (over 80 g daily for 40 years). In MRI examination, the tumor was isodense on T1-weighted image and partially round and hyperintense on T2-weighted image. Dynamic contrast-enhanced MRI with gadolinium ethoxybenzyl diethylenetriaminepentaacetic acid (EOB-MRI) was performed. In the arterial phase following contrast injection, the center of the tumor was enhanced intensely and homogeneously. In the delayed phase and hepatobiliary phase, the tumor was completely washed out (Figure 1), similar to findings in malignant tumors. It was difficult or impossible to differentiate IPT from HCC on EOB-MRI. The mass was clinically diagnosed as an HCC according to the 2008 guidelines of Japanese Society of Hepatology [11]. We attempted transcatheter arterial infusion of iopamiron and observed nodules in the right posterior lobe of the liver (Figure 2). We next attempted transcatheter arterial chemoembolization (TACE) with lipiodol (2 mL) plus epirubicin (20 mg) and miriplatin (70 mg) plus lipiodol (2 mL) plus porous gelatin particles (Gelpart). After TACE, partial lipiodol accumulation was observed. Moreover, we attempted radiofrequency ablation (RFA). 

**Figure 3:**
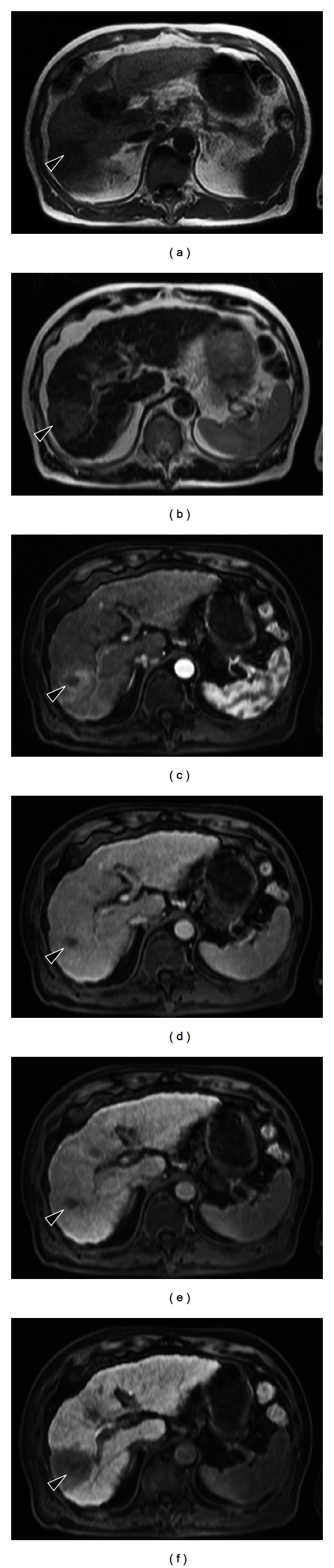
MR images obtained approximately two months after transcatheter arterial chemoembolization and radiofrequency ablation. (a) A hypodense area measuring approximately 3 cm in diameter is observed on T1-weighted image. (b) A hyperintense area is found on T2-weighted image. ((c)–(f)) Gadolinium ethoxybenzyl diethylenetriamine pentaacetic acid-enhanced images. In the arterial phase following contrast injection (c), the center of the tumor is enhanced intensely and homogeneously. In the delayed phase (d) and hepatobiliary phase (f), the tumor is completely washed out. e: portal phase.

**Figure 5:**
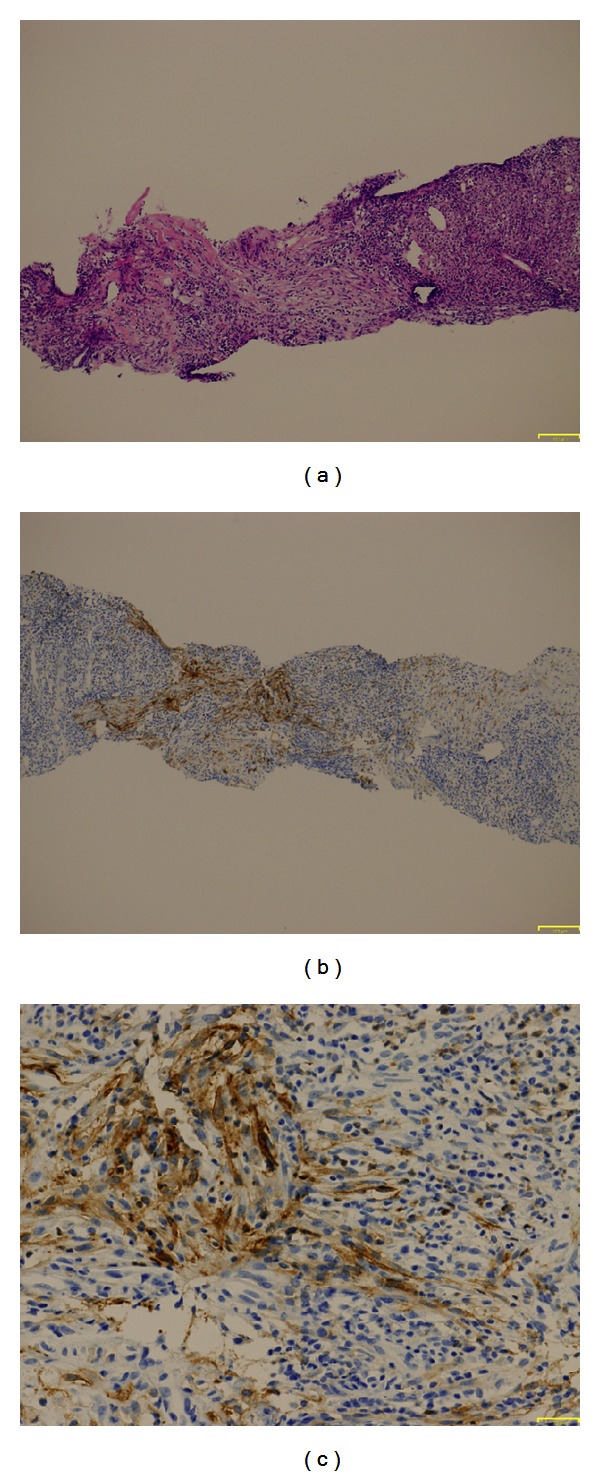
Pathological finding. (a) Liver biopsy of the hepatic mass shows extensive infiltration of lymphocytes and neutrophils, and fibrosis (hematoxylin and eosin staining, ×100). ((b) and (c)) Immunohistochemical staining shows CD10-positive spindle shaped myofibroblasts in a background of collagenous and fibrous tissue ((b): ×100, (c): ×400).

After 3 months, CT revealed a 3 cm mass located in the right posterior lobe of the liver showing early phase contrast enhancement and delayed phase washout with rim enhancement. MRI revealed that the mass was entirely hypointense on a plain T1-weighted image and hyperintense on plain T2-weighted image. On EOB-MRI, the tumor was enhanced intensely and homogeneously in arterial phase and entirely hypointense in the delayed phase and hepatobiliary phase (Figure 3). An FDP-PET scan confirmed abnormal metabolic activity with a high standardized uptake value of 7.3 in the lesion. We attempted percutaneous needle biopsy to differentiate between HCC, cholangiocarcinoma, metastatic liver tumor, and liver abscess. Histologically, the tumor consisted of infiltrated inflammatory cells comprising lymphoplasma cells, neutrophils, and eosinophils in a background of stroma composed of interlacing bundles of myofibroblasts and collagen bundles (Figure 5(a)). The surrounding liver parenchyma showed no evidence of cirrhosis. Immunohistochemical studies showed positive staining for CD10 (Figures 5(b) and 5(c)), SMA, and CD34 and negative staining for CD117. There was no immunostaining for IgG4 in the entire liver tissues. There were no findings of HCC.

**Figure 6:**
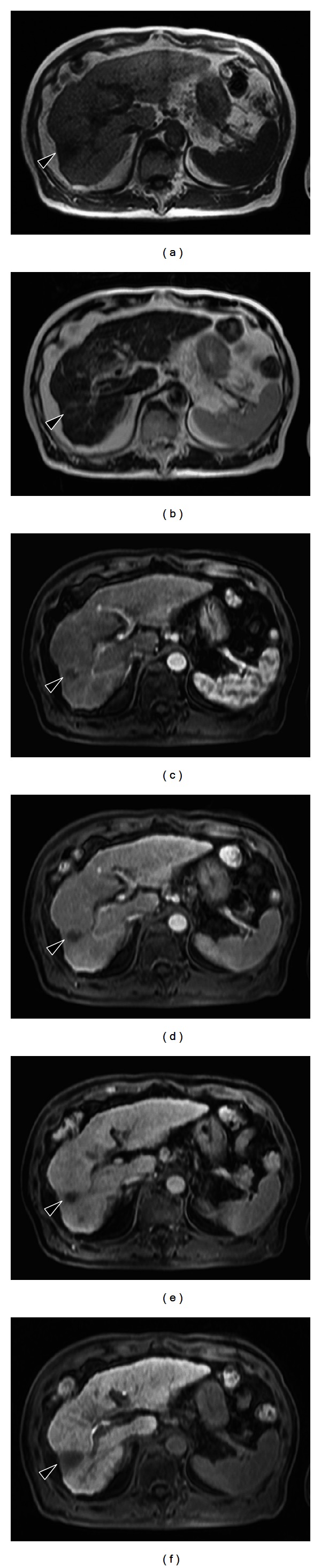
MR images obtained approximately four months after transcatheter arterial chemoembolization and radiofrequency ablation. (a) A hypodense area 1.5 cm in diameter is observed on T1-weighted image. (b) A hyperdense area is found on T2-weighted image. ((c)–(f)) Gadolinium ethoxybenzyl diethylenetriamine pentaacetic acid-enhanced images. In the arterial phase following contrast injection (c), the center of the tumor is enhanced intensely and homogeneously. In the delayed phase (d) and hepatobiliary phase (f), the tumor is completely washed out. e: portal phase. The tumor size has diminished compared to Figure 4.

A histopathological diagnosis of IPT was established. After the diagnosis, antibiotics were administered for one week. Two months after the diagnosis, the IPT was reduced spontaneously from 30 mm to 15 mm (Figure 6). 

## 3. Discussion

Certain bacteria including *Escherichia coli* [12], *Staphylococcus aureus* [13], and Gram-positive cocci [12] have been proposed as the causative agents of IPT of the liver. Based on the imaging findings, IPT is sometimes misdiagnosed as a malignant tumor such as HCC, cholangiocellular carcinoma (CCC) or metastatic liver cancer, or liver abscess [12, 14]. It is important to diagnose IPT by liver biopsy because the tumor can be cured by medical treatments including nonsteroidal antiinflammatory drugs, antibiotics, and steroids [14]. In the present case, liver biopsy of the hepatic mass showed extensive infiltration of lymphocytes and neutrophils as well as fibrosis, suggesting IPT. Therefore, we first treated the patient with antibiotics for one week. 

The IPT in our case was histologically composed of neutrophils, lymphocytes, plasma cells, and fibroblastic cells. Someren [15] classified IPT of the liver into three types: (i) hyalinized sclerosing; (ii) xanthogranuloma; (iii) plasma cell granuloma subtypes. However, previous reports [14] showed that histological appearance did not correlate with the disease stage and that these various morphological features were observed to some extent simultaneously even in the same case [16]. It has been speculated that the differences between these histological subtypes might reflect varying disease processes or periods. However, the presence of neutrophils in IPT has not been described. Strictly speaking, therefore, the present case cannot be classified into any of the subtypes. Although the tumor in our case cannot be strictly diagnosed as classical IPT of the liver, it is consistent with the broad diagnostic criteria.

Recently, IPT can be classified pathologically into two types: fibrohistiocytic and lymphoplasmacytic, from IgG4-related disease [17]. Fibrohistiocytic IPT is characterized by xanthogranulomatous inflammation, multinucleated giant cells, and neutrophilic infiltration and occurs predominantly in the peripheral hepatic parenchyma as mass-forming lesion. In contrast, lymphoplasmacytic IPT shows diffuse lymphoplasmacytic infiltration and prominent eosinophilic infiltration and is found exclusively around the hepatic hilum. In addition, venous occlusion with little inflammation and cholangitis without periductal fibrosis are frequently observed in the fibrohistiocytic type, whereas obliterative phlebitis and cholangitis with periductal fibrosis are common features of the lymphoplasmacytic type. Interestingly, IgG4-positive plasma cells are significantly more numerous in the lymphoplasmacytic than fibrohistiocytic type. Immunohistochemically, our case showed IgG-negative plasma cells and may be classified into the fibrohistiocytic type. 

In the present case, findings observed in the liver biopsy, especially the focal CD10 positivity and neutrophilic infiltration, may suggest changes secondary to previous treatment. Heat injuries by RFA involving adjacent organs and Glisson's sheaths have been reported to cause complications such as cholecystitis [18]. In our case, hepatocellular damage from cirrhosis and RFA/TACE treatment might have given rise to the IPT or caused enlargement of the IPT. 

In conclusion, this paper reports for the first time the EOB-MRI and FDP-PET findings of IPT of the liver, which is difficult to differentiate from malignant tumor. In addition, our case may be a rare case of enlargement of hepatic IPT induced by TACE or/and RFA. IPT should be suspected when treatment for a suspected liver lesion, whether it is HCC or abscess, does not produce expected response. 

## Figures and Tables

**Figure 4:**
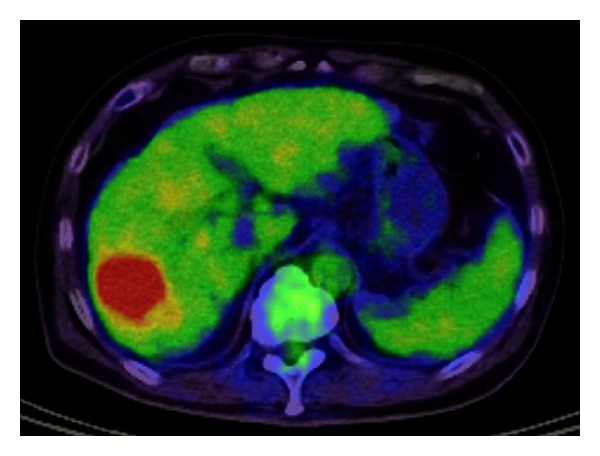
Positron emission tomography- (PET-) CT. Fluorine-18 fluorodeoxyglucose positron emission tomography (FDG-PET) shows abnormal uptake in segmental 6. The arrowhead denotes abnormal uptake lesion.
